# Percutaneous Management of Biliary Enteric Anastomotic Strictures: An Institutional Review

**DOI:** 10.7759/cureus.2228

**Published:** 2018-02-26

**Authors:** Muhammad Azeemuddin, Nauman Turab, Mustafa Belal H Chaudhry, Shoaib Hamid, Mohammad Hasan, Raza Sayani

**Affiliations:** 1 Department of Radiology, The Aga Khan University, Karachi.; 2 Radiology Department, Dow University of Health Sciences (DUHS), Karachi, Pakistan; 3 Radiology, Sultan Qaboos University; 4 Medical Student, Jinnah Sindh Medical University (SMC)

**Keywords:** stricture, biliary enteric anastomotic, percutaneous transhepatic balloon dilatation, iatrogenic bile duct injuries

## Abstract

Purpose

Stricture formation at the biliary enteric anastomotic site is a common complication due to fibrotic healing. Few therapeutic options are available for biliary-enteric anastomotic site stricture (BES) including new surgical reconstruction or percutaneous transhepatic biliary drainage followed by balloon dilation of BES or stent placement. The purpose of this study is to assess the technical success, complications and reintervention rate of percutaneous transhepatic balloon dilatation (PTBD) of BES after iatrogenic bile duct injuries (BDI).

Methods

A retrospective review of patients who underwent PTBD for benign resistant BES, previously treated for iatrogenic BDI, from December 2004 to January 2016 was performed. Diagnostic transhepatic cholangiogram was performed to assess the level of obstruction. BES was dilated using 8-12 mm diameter balloons followed by placement of eight to ten Fr internal-external drainage catheters, which were removed after three to six weeks post-PTBD cholangiogram. Follow-up by clinical assessment, liver function tests, and ultrasound was done. Fischer exact test was used to determine if there was a significant association between PTBD sessions and recurrent strictures.

Results

In total, 37 patients underwent 66 sessions of PTBD, including 10 (27%) males and 27 (73%) females. The mean age was 41.3 years (range 23–70 years). Out of these, 29 (78%) were treated with choledochojejunostomy and eight (22%) with hepaticojejunostomy. 100% technical success was achieved in all the PTBD sessions. Nineteen (51.3%) patients were treated with a single PTBD session. Mean follow-up time was 36 months (range 1–75 months). Eighteen (48.7%) patients needed reintervention, out of these, 11 (29.7%) were symptom-free after second session on three-year follow-up, three (8%) were symptom-free after the third session of PTBD. No significant difference was observed in risk of recurrent strictures after first and second PTBD sessions [18 (48%) vs. 7 (39%); p-value 0.495]. In four (11%) patients, the symptoms persisted and BES recurred even after third session and those were treated by placing metallic stent. In total, three (8.1%) patients got complicated with the stone formation; in two (5%) patients stone was successfully removed percutaneously and in one (3%) patient percutaneous attempt failed so it was followed by surgical removal.

Conclusion

PTBD is a safe and useful treatment option for benign BES for long-term symptom-free time-period. However, there is no significant difference in developing recurrent BES after PTBD sessions. Few patients with resistant strictures might require stent placement.

## Introduction

Laparoscopic cholecystectomy is one of the most commonly performed elective surgical procedures in the world for symptomatic and asymptomatic cholelithiasis [1]. The laparoscopic surgical approach is overall safe with reduced recovery time and lower rate of complications than traditional open approach. However, there is reported incidence of 0.1 to 0.2% iatrogenic bile duct injuries (BDI) with open cholecystectomy which further increases to 0.4 to 0.6% with laparoscopic cholecystectomy [2-4]. Most of the repair procedures include biliary-enteric (BE) anastomoses which is technically challenging [5]. Other common reasons for BE anastomosis are resection of malignancies of gallbladder, pancreas, and in cholangiocarcinoma. The reparative procedures often offer the best long-term results with considerably high clinical success rates between 80% and 92% [6]. The benign anastomotic stricture formation at the biliary-enteric anastomotic site is the most common complication, due to fibrotic healing, which can occur in 7–23% cases [7,8]. The biliary-enteric anastomotic stricture (BES) can further lead to obstructive jaundice, recurrent cholangitis with the risk of secondary biliary cirrhosis and portal hypertension. Overall few therapeutic options are available for BES, including new surgical reconstruction or percutaneous transhepatic biliary drainage followed by balloon dilation. Although surgical reconstruction is considered most definitive treatment but the morbidity and mortality rates are high and the recurrence rate is 20%–25%. The alternative to surgery is percutaneous transhepatic balloon dilatation (PTBD).

In recent years, PTBD is increasingly used to evaluate and treat such patients with a quick recovery and far less morbidity. However, there is a paucity of local data that describes clinical outcomes and re-intervention rate of patients with recurrent BES after repair of iatrogenic BDI [9,10]. Therefore, the purpose of this study is to assess the technical success, complications, and reintervention rate of PTBD in BES after iatrogenic BDI.

## Materials and methods

A retrospective cross-sectional study was conducted in Section of Vascular and Interventional Radiology (VIR), Department of Radiology, after the institutional ethical review committee approval. The requirement for informed consent from the patient or next of kin for participation in the study was waived. All patients who underwent PTBD for benign resistant BES, previously treated for iatrogenic BDI, from December 2004 to January 2016 were included. Patients of pediatric age group (<16 years), malignant BES and known history of hepatobiliary malignancy were excluded. The data sources included picture archiving and communication system (PACS), radiology information system (RIS) and confidential file record (CFR). Images were reviewed through PACS, radiology reports and records were reviewed through RIS, and CFR was used to review clinical notes and surgical/procedural notes. All the procedures were performed by one out of five interventional radiologists (IR) with variable experience of three to fifteen years. These IRs directly supervised or were assisted by seven VIR fellows during the study period.

All the patients referred to VIR with chief complaints of obstructive jaundice or to rule out BES, after repair of iatrogenic bile duct injury, were clinically assessed in VIR clinic. Those with clinical and laboratory signs and symptoms of obstructive jaundice underwent pre-intervention imaging which included ultrasonography and/or computed tomography (CT) scan abdomen with standard departmental protocols. Subsequently, percutaneous transhepatic cholangiogram (PTC) was performed to assess the bile flow and level of obstruction in patients with biliary dilatation on imaging. The cases with biliary obstruction or BES were offered PTBD procedure, after discussing with the primary physician.

All the PTBD procedures were performed on the elective basis and followed our routine departmental protocol which included correction of coagulopathy, i.e. platelet counts of >75 x 10^3^/microliter and international normalization ratio (INR) of <1.5, prior to PTBD procedure, administration of broad-spectrum antibiotic and acquisition of informed consent. Prophylactic intravenous antibiotics (1 g ceftriaxone or 4.5 g piperacillin/tazobactam) were administered during an hour before the procedure in all patients. Physiological vitals monitoring was routinely carried out during all the procedures as per protocol.

**Figure 1:**
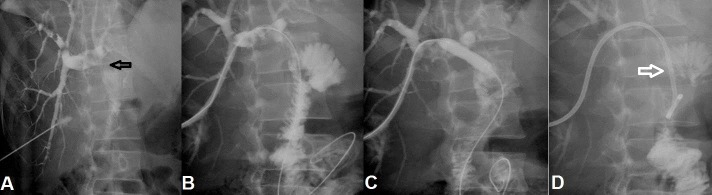
A 45-year-old male with iatrogenic common bile duct injury during cholecystectomy underwent primary choledochojejunostomy. (A) The PTC shows a tight stricture (black arrow) at biliary enteric anastomotic site. (B) Biliary enteric anastomotic site was negotiated using wire and advanced into the bowel. (C) Balloon dilatation of tight biliary enteric stricture was performed. (D) Subsequently, a 10 Fr internal-external biliary drain (white arrow) was placed across biliary enteric anastomotic stricture as safety. PTC: Percutaneous transhepatic cholangiogram.

PTBD was performed under local anesthesia with mild to moderate sedation as per requirement using a combination of intravenous fentanyl citrate and midazolam. The intrahepatic biliary duct (right or left) was punctured using 18 or 20 gauge access needle and a baseline cholangiogram was obtained. After performing the PTC, in stable patients, the procedure was proceeded to cross the BES in the same session. It was followed by placing a seven Fr vascular access sheath (Terumo Corp, Tokyo, Japan) over a 0.038-inch hydrophilic-coated guide wire. Subsequently, using five Fr vertebral catheter (Terumo Corp, Tokyo, Japan), the 0.038-inch guide wire was exchanged with an Amplatz wire (Cook Inc, Bloomington, IN). The BES was dilated with angioplasty balloon (8–12 mm diameter, Boston scientific) maximum up to 14 atmosphere pressure for a period of two to three minutes. Maximum dilatation was performed thrice in a single session. It was followed by a cholangiography each time to assess the response and, finally 8–12 Fr internal/external biliary drainage catheter (IEBC, Cook Medical) was placed with its tip across the BES into the small bowel. Later on, the patient was discharged after gaining satisfactory clinical and vital stability with IEBC capped and internalized (Figure 1).

If the patient was unstable in terms of vitals or acute cholangitis, either 8 Fr or 10 Fr external biliary drainage catheter (EBC) was placed in the dilated biliary system and further manipulation was planned after two days, so that the patient's general condition and infection (if any) gets improved. The decision always depended on the patient’s clinical status and at the discretion of interventional radiologist. Thereafter, the preexisting EBC was exchanged for a sheath and PTBD session was performed in a similar way described above.

**Figure 2:**
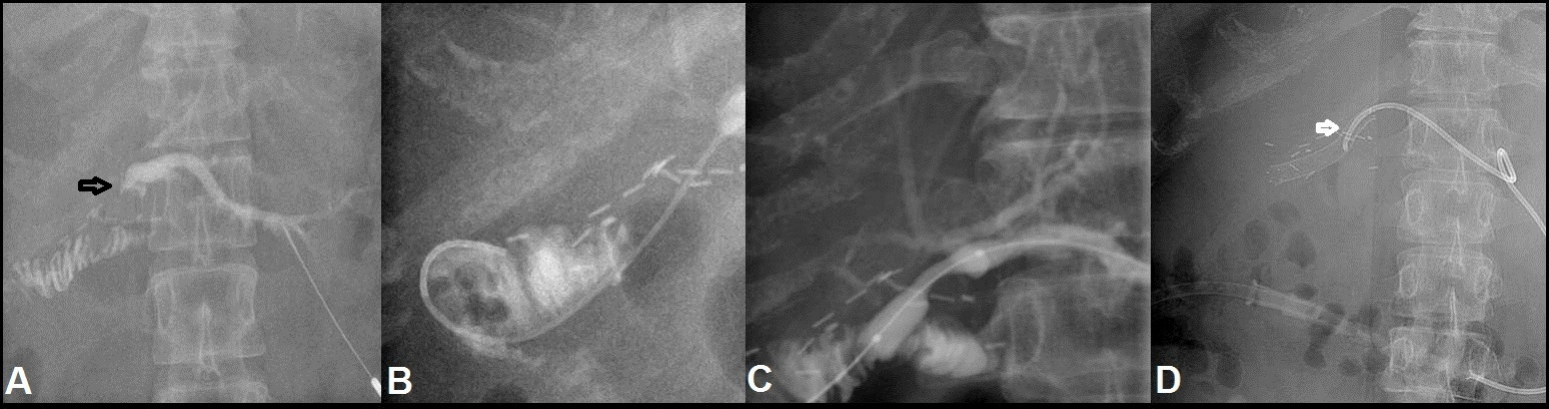
A 37-year-old female with iatrogenic CBD injury during laparoscopic cholecystectomy, initially managed conservatively, followed by hepaticojejunostomy, developed recalcitrant BES. (A) Underwent PTC showing significant narrowing at the hepaticojejunostomy anastomotic site (black arrow) with minimal dilatation of intrahepatic biliary radicals. (B) Subsequently, a guide wire is negotiated from the site of stricture. (C) It is followed by conventional balloon being employed for dilation demonstrating persistent shouldering at the stricture site. (D) This is followed by stent placement (white arrow) at the site of stricture. BES: Biliary-enteric anastomotic site stricture; CBD: Common bile duct; PTC: Percutaneous transhepatic cholangiogram.

If the procedure was successful technically and the patient was asymptomatic during the interval period with normalization of laboratory parameters compared with values before the PTBD session, then six weeks follow-up cholangiogram was performed through the IEBC while pulling it out to assess the patency of the BES. If the biliary flow had restored with the resolution of obstruction then the IEBC was removed and the patient was followed up at the clinic after six weeks. In case of persistence of BES, repeat PTBD was performed with the similar protocol and the IEBC was repositioned with its tip distal to BES for further two to six weeks. It was followed by PTC to assess the effect of treatment. In the event of further failure or recurrence of BES, the PTBD session was repeated. After maximum three unsuccessful attempts, the patients were offered the option of surgical revision or stenting. In cases of biliary stent placement, the IEBC was placed and then removed after three to five days after post-stenting cholangiogram, if the stent was patent (Figure 2).

All the procedures were considered technically successful if there was fluent passage of injected contrast through BE anastomosis into the small bowel. The duration of BE anastomosis patency was defined as the time between removal of the EBC/IEBC till documentation of BES recurrence on a subsequent PTC, at patient’s death, or completion of the data collection period. On every discharge from the hospital, the patient was advised to follow up in VIR clinic if they had any recurrence of symptoms like jaundice and pruritus.

Patients were clinically followed up in VIR clinic by laboratory parameters including specific liver function profile, total leukocyte counts, and blood coagulation profile at six weeks, three, six, and twelve months after the procedure. Ultrasonography was performed at six weeks and then after every six months on follow-up. The clinical success was considered if there were absence of biliary obstructive symptoms in conjunction with normal laboratory parameters and absence of intrahepatic biliary dilatation on ultrasonography. If the patient had clinical and imaging signs of obstruction or BES then the patient was managed according to the same departmental protocol detailed above.

Data was entered and analysis was performed using SPSS version 20 (IBM Corporation). Simple descriptive analysis was performed and reported as frequencies with percentages for categorical variables and means with range for continuous variables. Fischer exact test was used to determine if there was a significant association between PTBD sessions and recurrent strictures. A p-value of <0.05 was considered statistically significant.

## Results

Total 37 patients were referred to VIR from general surgery department who were previously treated for iatrogenic BDI. Ten (27%) patients were males and twenty-seven (73%) were females with mean age of 41.3 years (range 23-70 years). In total, eight (22%) patients were treated with hepaticojejunostomy and the rest twenty-nine (78%) were treated with choledochojejunostomy. These 37 patients underwent total 66 sessions of PTBD. The 19 (52%) patients in total were treated successfully after the first PTBD session. The remaining 18 (48%) patients had the recurrence and underwent second PTBD session. Eleven (30%) patients were symptom-free after second PTBD session for BES on three-year follow-up. However, seven (19%) patient had the recurrence. Out of these seven patients, three (8.1%) patients became symptom-free after the third PTBD session for BES. In four (11%) out of 37 patients, the symptoms persisted and the BES recurred even after the third PTBD session. All of them then further managed by placing a metallic biliary stent, after discussing with the surgical team. No statistically significant difference was seen in developing recurrent BES after PTBD sessions.

All the patients were followed up for an average time of 36 months (1-75 months). Two (5%) patients were lost to follow-up after first PTBD session, one each after the second and third sessions. Eleven (30%) patients were symptom-free after second PTBD session on three-year follow-up and two (5%) patients were symptom-free for two years after the third PTBD session. Out of these 37 patients, three (8%) patients got complicated with the intrahepatic biliary stone formation. In two of them, the stone was successfully removed percutaneously. However in one (3%) patient the percutaneous attempt failed due to the multiplicity of calculi and they were removed surgically.

## Discussion

Iatrogenic biliary ductal injury is rare but not uncommon. The subsequent surgical repair can result in local stricture formation in 10% to 30% of the cases [8,11]. These recurrent strictures can be challenging to manage due to significant local fibrosis. Pitt, et al. and Pellegrini, et al. have reported up to 68% stricture recurrence within three years and up to 80% recurrence at BE anastomotic site [7,12]. The overall frequency of BE anastomosis has increased in the recent years due to increase in the volume of hepatobiliary surgery [13]. The management of BES continues to be a challenging problem and requires a multidisciplinary approach. Surgical repair not only increases the healthcare costs but also carries substantial morbidity and mortality. Non-surgical approaches for BES repair are a time-tested option and achieve good results with minimal morbidity and mortality in most patients.

The use of percutaneous techniques to treat BES was first reported in 1974 by Molnar and Stockum [14]. A total of 90% of patients with benign strictures can be treated with a percutaneous approach (i.e., IEBC placement, conventional or cutting balloon PTBD or stent placement). Goykhman, et al. treated BES in 15 patients with conventional PTBD and successfully managed 47% patients over a mean follow-up duration of 24 months [15]. Similarly, Misra, et al. evaluated 51 patients who were treated with conventional PTBD and the procedure was successful in 58.8% patients over a mean follow-up duration of 76 months [10]. In the past decade, a number of researchers (Saad, et al.; Atar, et al. and Kakani, et al.) have evaluated the use of cutting balloon in treating the BES [16-18]. All of these studies had smaller sample size than our study with a primary technical success rate of 50%–100% and primary patency rate from 10 months to 24 months. This emerging technology has offered easier dilation and expected improved long-term patency. Recently, Lee, et al. and Mukund, et al. have successfully used the combination of cutting and conventional balloons to perform PTBD [19,20]. Lee did not report the technical success, but Mukund reported 100% success in a small sample size of eight patients with mean symptom-free duration of 14 months on follow-up.

Labelling a procedure as a technical failure was based on anecdotal evidence. Mukund, et al. kept a three-week interval between two sessions, and labelled it as a failure if BES persisted after third session [20]. In contrast, we kept a three- to six-week interval between two sessions and labelled it as a failure if the BES persisted after three PTBD sessions. We achieved 100% technical success rate in all 66 PTBD sessions we performed only using conventional balloons. One reason which could have resulted in this was dilating a balloon near to its burst pressure, keeping it inflated for up to two to three minutes and dilating a BES up to three times in a single session. We also did cholangiogram after every dilatation in a single setting to assess the effect of PTBD or to rule out any iatrogenic perforation. Our results are more promising than previously reported failure rate of up to 33% [9,21]. Our technical success rate with conventional PTBD is comparable to the success rate of combined conventional and cutting PTBD and only cutting PTBD [15-20]. We did not use cutting balloons in contrast to Mukund, et al, Atar, et al. and Kakani, et al., even without using that our patients had mean symptom-free duration of 36 months which is relatively better than any of those studies.

There were certain limitations to our study. First, it was a retrospective study and all the related shortcomings in data collection should be considered like loss to follow-up; this could have affected our results. Second, we omitted to assess and analyze the pre-PTBD, post-PTBD and follow-up laboratory workup, due to its partial unavailability in more than half of the cases. Third, our sample size was relatively small with mean follow-up duration of only 36 months. Fourth, we did not have cutting balloons available, so we were left with the option of percutaneous biliary stent placement to manage recalcitrant BES. There is a possibility that the use of cutting balloon could have ruled out the need of using metallic stent in such cases. Here is a need of a prospective cohort study or blinded randomized control trial to validate the reported outcomes related to the individual and combined use of both conventional and cutting balloons.

PTBD with conventional balloons is a safe and useful treatment option for benign BES for long-term symptom-free time-period. However, there is no significant difference in developing recurrent BES after PTBD sessions. Few patients with recalcitrant strictures might require stent placement.

## Conclusions

In conclusion, iatrogenic biliary ductal injury is rare but not uncommon. The subsequent surgical repair can result in local stricture formation. These recurrent strictures can be challenging to manage due to significant local fibrosis.

PTBD with conventional balloons is a safe and useful treatment option for benign BES for long-term symptom-free time-period.

We present our experience and results in treating these strictures. However, there is no significant difference in developing recurrent BES after PTBD sessions. Few patients with recurrent strictures might require stent placement.
